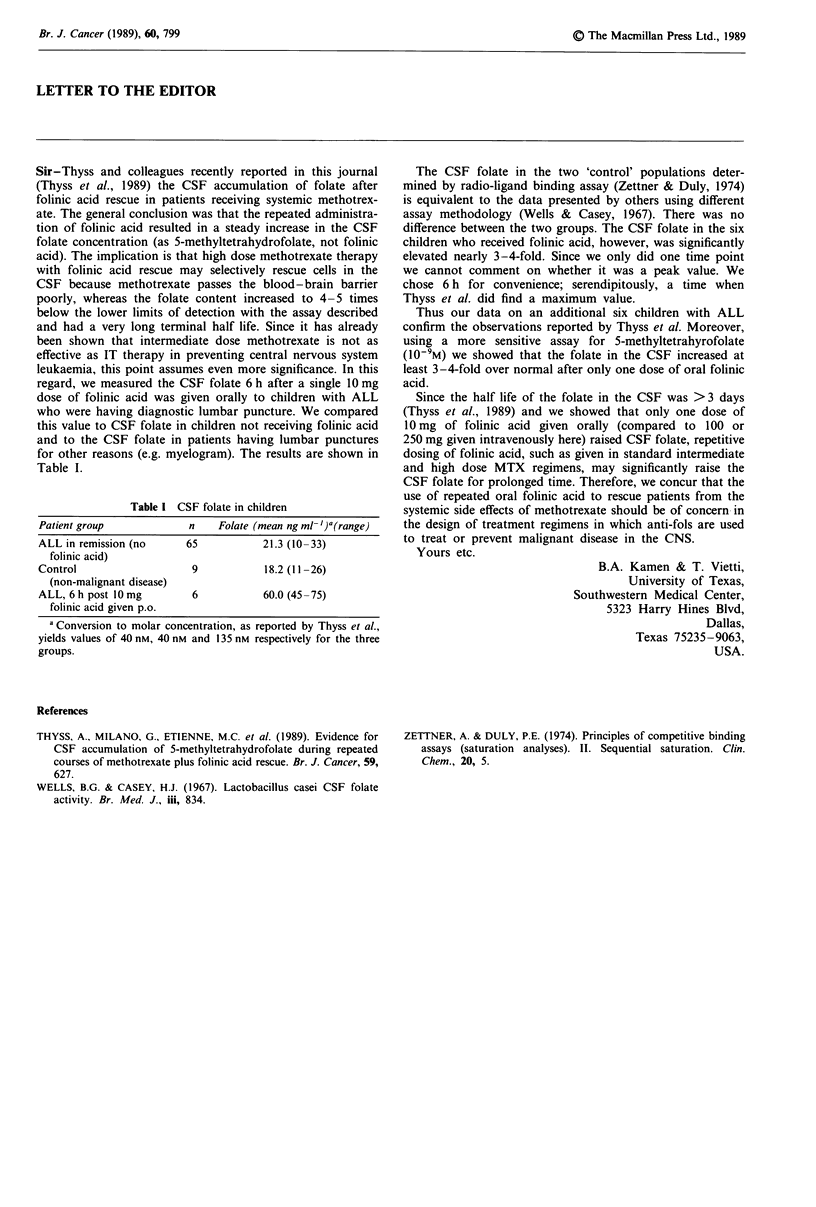# Evidence for CSF accumulation of 5-methyltetrahydrofolate during repeated courses of methotrexate plus folinic acid rescue.

**DOI:** 10.1038/bjc.1989.363

**Published:** 1989-11

**Authors:** B. A. Kamen, T. Vietti


*Br. J. Cancer* (1989), **60**, 799

© The Macmillan Press Ltd., 1989

### LETTER TO THE EDITOR

**Sir**–Thyss and colleagues recently reported in this journal (Thyss *et al.*, 1989) the CSF accumulation of folate after folinic acid rescue in patients receiving systemic methotrexate. The general conclusion was that the repeated administration of folinic acid resulted in a steady increase in the CSF folate concentration (as 5-methyltetrahydrofolate, not folinic acid). The implication is that high dose methotrexate therapy with folinic acid rescue may selectively rescue cells in the CSF because methotrexate passes the blood–brain barrier poorly, whereas the folate content increased to 4–5 times below the lower limits of detection with the assay described and had a very long terminal half life. Since it has already been shown that intermediate dose methotrexate is not as effective as IT therapy in preventing central nervous system leukaemia, this point assumes even more significance. In this regard, we measured the CSF folate 6 h after a single 10 mg dose of folinic acid was given orally to children with ALL who were having diagnostic lumbar puncture. We compared this value to CSF folate in children not receiving folinic acid and to the CSF folate in patients having lumbar punctures for other reasons (e.g. myelogram). The results are shown in Table I.

**Table I** CSF folate in children

| *Patient group* | *n* | *Folate (mean ng ml$^{-1}$)$^a$(range)* |
|---|---|---|
| ALL in remission (no folinic acid) | 65 | 21.3 (10–33) |
| Control (non-malignant disease) | 9 | 18.2 (11–26) |
| ALL, 6 h post 10 mg folinic acid given p.o. | 6 | 60.0 (45–75) |

[a] Conversion to molar concentration, as reported by Thyss *et al.*, yields values of 40 nM, 40 nM and 135 nM respectively for the three groups.

The CSF folate in the two 'control' populations determined by radio-ligand binding assay (Zettner & Duly, 1974) is equivalent to the data presented by others using different assay methodology (Wells & Casey, 1967). There was no difference between the two groups. The CSF folate in the six children who received folinic acid, however, was significantly elevated nearly 3–4-fold. Since we only did one time point we cannot comment on whether it was a peak value. We chose 6 h for convenience; serendipitously, a time when Thyss *et al.* did find a maximum value.

Thus our data on an additional six children with ALL confirm the observations reported by Thyss *et al.* Moreover, using a more sensitive assay for 5-methyltetrahyrofolate ($10^{-9}$M) we showed that the folate in the CSF increased at least 3–4-fold over normal after only one dose of oral folinic acid.

Since the half life of the folate in the CSF was >3 days (Thyss *et al.*, 1989) and we showed that only one dose of 10 mg of folinic acid given orally (compared to 100 or 250 mg given intravenously here) raised CSF folate, repetitive dosing of folinic acid, such as given in standard intermediate and high dose MTX regimens, may significantly raise the CSF folate for prolonged time. Therefore, we concur that the use of repeated oral folinic acid to rescue patients from the systemic side effects of methotrexate should be of concern in the design of treatment regimens in which anti-fols are used to treat or prevent malignant disease in the CNS.

Yours etc.

B.A. Kamen & T. Vietti,
University of Texas,
Southwestern Medical Center,
5323 Harry Hines Blvd,
Dallas,
Texas 75235–9063,
USA.

### References

THYSS, A., MILANO, G., ETIENNE, M.C. *et al.* (1989). Evidence for CSF accumulation of 5-methyltetrahydrofolate during repeated courses of methotrexate plus folinic acid rescue. *Br. J. Cancer*, **59**, 627.

WELLS, B.G. & CASEY, H.J. (1967). Lactobacillus casei CSF folate activity. *Br. Med. J.*, **iii**, 834.

ZETTNER, A. & DULY, P.E. (1974). Principles of competitive binding assays (saturation analyses). II. Sequential saturation. *Clin. Chem.*, **20**, 5.